# Measuring anxiety and depression in parents of hospitalized children during the COVID-19 pandemic in a pediatric Italian hospital

**DOI:** 10.1192/j.eurpsy.2021.1787

**Published:** 2021-08-13

**Authors:** C. Correale, I. Tondo, C. Falamesca, G. Amodeo, F. Boldrini, T. Grimaldi Capitello, F. Vigevano, S. Cappelletti

**Affiliations:** 1 Clinical Psychology, Bambino Gesù Children Hospital, Rome, Italy; 2 Neurological Sciences, Bambino Gesù Children Hospital, Rome, Italy

**Keywords:** COVID-19 pandemic, parents, Hospitalized children, anxiety and depression

## Abstract

**Introduction:**

Parents of hospitalized children with chronic illness (HCCI) during the COVID-19 epidemic may face huge pressure and worry, leading to mental health issues. Parent’s depression and anxiety disorders increase the risk of mental health problems in the child and affect his/her recovery.

**Objectives:**

The aim of this study was to assess the prevalence rate of depressive and anxiety symptoms among a pilot sample of parents of HCCI (in- and out-patients) with diagnosis of epilepsy (9), cystic fibrosis (8) and congenital heart anomalies (6) during COVID-19 pandemic. Pediatric patients were under a regular Children Hospital medical and psychological follow-up program.

**Methods:**

We conducted a cross-sectional study among 23 Italian parents (15 F; 8 M) of HCCI during the COVID-19 epidemic period. We performed face-to face interviews and assessed depressive and anxiety symptoms with the Patient Health Questionnaire (PHQ-9) and the Generalized Anxiety Disorders (GAD-7) questionnaire during scheduled follow up visits.

**Results:**

The anxiety score of parents of HCCI was 4.43 ± 3.17, of which 39.1% of parents were anxious (≥5 points), while the depression score was 4.04 ± 2.67, of which 30.4% of parents show depressive symptoms (≥5 points). The prevalence of comorbid depressive and anxiety symptoms was 26.1% among the entire sample.

**Conclusions:**

Preliminary data of our pilot study showed a high prevalence of anxious depressive symptoms and comorbidity among parents of HCCI. Timely provision of psychologic interventions are needed during and after COVID-19 pandemic in order to empower parenting and promote children recovery and quality of life.

**Disclosure:**

No significant relationships.

